# Development of secure infrastructure for advancing generative artificial intelligence research in healthcare at an academic medical center

**DOI:** 10.1093/jamia/ocaf005

**Published:** 2025-01-21

**Authors:** Madelena Y Ng, Jarrod Helzer, Michael A Pfeffer, Tina Seto, Tina Hernandez-Boussard

**Affiliations:** Department of Medicine, Stanford University, Stanford, CA 94305, United States; Technology and Digital Solutions, Stanford Health Care, Stanford, CA 94305, United States; Department of Medicine, Stanford University, Stanford, CA 94305, United States; Technology and Digital Solutions, Stanford Health Care, Stanford, CA 94305, United States; Technology and Digital Solutions, Stanford Health Care, Stanford, CA 94305, United States; Department of Medicine, Stanford University, Stanford, CA 94305, United States; Department of Biomedical Data Sciences, Stanford University, Stanford, CA 94305, United States; Department of Surgery, Stanford University, Stanford, CA 94305, United States

**Keywords:** large language models, artificial intelligence, healthcare informatics infrastructure, security, privacy, large-scale research, technology adoption

## Abstract

**Background:**

Generative AI, particularly large language models (LLMs), holds great potential for improving patient care and operational efficiency in healthcare. However, the use of LLMs is complicated by regulatory concerns around data security and patient privacy. This study aimed to develop and evaluate a secure infrastructure that allows researchers to safely leverage LLMs in healthcare while ensuring HIPAA compliance and promoting equitable AI.

**Materials and Methods:**

We implemented a private Azure OpenAI Studio deployment with secure API-enabled endpoints for researchers. Two use cases were explored, detecting falls from electronic health records (EHR) notes and evaluating bias in mental health prediction using fairness-aware prompts.

**Results:**

The framework provided secure, HIPAA-compliant API access to LLMs, allowing researchers to handle sensitive data safely. Both use cases highlighted the secure infrastructure’s capacity to protect sensitive patient data while supporting innovation.

**Discussion and Conclusion:**

This centralized platform presents a scalable, secure, and HIPAA-compliant solution for healthcare institutions aiming to integrate LLMs into clinical research.

## Background

The use of generative artificial intelligence (AI) tools such as large language models (LLMs) in healthcare has the potential to improve patient care, alleviate clinical burden, and enhance operational efficiency. Despite these upsides, leveraging LLMs in healthcare is complicated due to complex regulations, including data security and patient privacy.^1^ Researchers often fine-tune existing pre-trained LLMs (eg, GPT, LLaMA, and PaLM) with clinical data, but these adjusted models become more susceptible to security threats and privacy leakage attacks.^2^ Furthermore, data pre-processing decisions vary drastically across research teams, which can lead to integrating sensitive or protected health information (PHI) into LLMs, creating security, privacy, and regulatory risks.^3^ Thus, academic medical centers^4^^,^^5^ are investing in secure infrastructure to train the next generation of researchers and learners, thereby enabling clinical LLM experimentation to scale safely and meet the pace of innovation. In addition, these institutions have also begun integrating HIPAA-compliant AI platforms. However, these implementations often lack the granular control over infrastructure security that is crucial for healthcare settings. Our approach offers a fully private deployment with Azure’s ExpressRoute, ensuring that no sensitive data is exposed to public internet channels, which distinguishes it from other platforms.

In January 2024, our healthcare institution launched “Secure GPT” chat web application for our research community to query GPT 4.0 with PHI in a closed system. The Secure GPT web app operates in a secured cloud environment to ensure no data passed through is retained or modifies the model. However, its manual single prompt approach did not meet researcher or learner needs. To address this, we developed a secure pathway to the OpenAI application programming interface (API), to enable our community to seamlessly access and integrate Secure GPT’s capabilities to create diverse, multi-modal healthcare use cases. To further safeguard this gateway from internal and external risks, we describe the security measures undertaken to ensure a secure environment for generative AI research, aiming to share our work and foster innovation in healthcare and beyond.

## Methods

Stanford Medicine’s information technology department, Technology and Digital Solutions (TDS), implemented a secure infrastructure for medical researchers, clinicians and in-house application developers to have private API-enabled endpoints to a dedicated Azure OpenAI Studio deployment. The models available in the deployment (at the time of writing) include GPT-4o mini, GPT-4o, GPT-4, GPT-4-32k, GPT-3.5-Turbo, text-embedding-ada-002, and DALL-E 3. The infrastructure team can deploy models on demand for each instance. In addition to using proprietary OpenAI models, our platform integrates various open-weight models, such as TinyLlama and Llama-2, within the same secure infrastructure. These models were accessed through the private Azure OpenAI service to maintain HIPAA compliance and ensure seamless integration of external models while safeguarding patient data.

To establish the secure infrastructure, TDS created a dedicated Azure subscription for hosting OpenAI Studio, supported by an existing business associate agreement (BAA) with Microsoft Azure and Stanford Health Care. This subscription can hold up to 30 OpenAI instances per region, with various tokens per minute (TPM) limits depending on the LLM used and limits granted. For example, a total of 10 million TPM/60 000 requests per minute could be allowed for the GPT-4o model in a subscription. Each instance has an initial call rate limit of 3000 TPM, adjustable upon request. As proof of concept, TDS configured an OpenAI instance for one research lab, providing a private URL endpoint and a secure API key stored in HashiCorp’s Vault to ensure privacy and security.

Two Azure ExpressRoute circuits establish connectivity that securely extend the on-premise network into Microsoft cloud services via a private connection, ensuring data does not travel via the public internet. This setup not only enhances security, but it also provides fast and reliable connection with consistent low latency. Private IP addresses and their corresponding names are registered in the School of Medicine's directory. This allows Stanford Health Care's Azure endpoints to be accessed securely from the School of Medicine and through both organizations' private networks. With the private endpoint IP address hosted on Stanford's network, any data submitted to the models stays within a secure and private environment. This ensures that sensitive information remains protected and accessible only within the approved network.

This secure infrastructure was developed through collaboration across the institution, including cloud engineering, security, and networking teams. By providing a secure, centrally managed environment, individual research labs avoid building their own microprocesses, reducing security risks associated with decentralized management. The cost of utilizing the API can be supported through research grants, institutional contributions, or in-kind support. However, clear policies and strategies are essential to ensure equitable and sustainable access given the variability in computational demands. Access is granted to researchers after Institutional Review Board (IRB) approval is obtained. The principal investigator is responsible for managing the API use among their team based on their research specifications. Institutional resources are available to assist with endpoint management (eg, token usage), reducing the technical burden on researchers.

To complete the secure infrastructure, researchers utilize a private GitLab for code source control and versioning, rather than the public GitHub. All software and systems within this infrastructure are managed and maintained by the cloud engineering team. TDS has since made similar connections to Google AI and Meta AI, offering Gemini 1.5-pro and Llama 3.1, respectively, to further enhance LLM experimental flexibility at our organization. By integrating all components into the TDS toolchain, a comprehensive, secure solution for researchers, clinicians and staff to access generative AI models efficiently and safely could be obtained.

## Use cases

To demonstrate the system's effectiveness, we present two early adopter use cases that involved securely accessing and processing patient data through Stanford Health Care’s Azure endpoints. IRB approval was obtained to ensure compliance with ethical standards and protect patient privacy.

### Use case: fall detection using Secure GPT API

To improve fall detection capabilities in healthcare settings, our team leveraged Stanford’s private API for Secure GPT to assess the effectiveness of LLMs, including GPT-4, in detecting post-operative fall events from electronic health records (EHR) clinical notes. This use case focused on processing clinical notes from a cohort of surgical patients, extracting sentences mentioning falls and their context using regex protocols. By conducting zero-shot and few-shot prompting, we evaluated the model's performance on a random sample of notes. The models demonstrated superior capabilities in detecting falls compared to traditional NLP methods, which have shown only mediocre discrimination capabilities. This approach exemplifies how the API can process clinical notes effectively, ensuring data privacy while leveraging advanced LLM capabilities to enhance clinical outcomes. Our findings were further validated in an external healthcare setting, highlighting the potential for scalable and secure AI integration in clinical practice.^6^

Our fall detection use case demonstrated the capabilities of Secure GPT in processing clinical notes. By leveraging GPT-4 and advanced regex protocols, the system provided faster and more accurate identification of fall-related events compared to traditional NLP methods. In addition, the secure infrastructure ensured that sensitive patient data was processed entirely within our protected network, maintaining full compliance with HIPAA regulations.

### Use case: evaluating bias in mental health analysis with Secure GPT API

Utilizing the Secure GPT API, we assessed the performance and bias of different LLMs across eight mental health conditions using clinical notes obtained from the EHR. We integrated ten LLMs of varying sizes, including TinyLlama-1.1B, Gemma-2B, Phi-3-mini, Gemma-7B, Llama-2-7B, Llama-2-13B, MentaLLaMA-7B, MentaLLaMA-13B, Llama-3-8B, and GPT-4. We evaluated each model’s performance and fairness in tasks such as depression and stress prediction. We employed fairness-aware prompts to reduce biases and analyzed results for demographic disparities. Domain-specific models outperformed larger LLMs, with GPT-4 achieving the best balance between performance and fairness. Fairness-aware prompts were effective in reducing bias without significantly compromising performance. The secure infrastructure and API facilitated a comprehensive evaluation of biases in LLMs, advancing equitable AI research and underscoring the importance of targeted prompting strategies and secure data handling.^7^

The secure infrastructure of Secure GPT was critical for this evaluation, as it allowed us to safely handle sensitive patient data and analyze model outputs in compliance with HIPAA regulations. The flexibility of the platform also enabled the integration of open-weight models, allowing for broader experimentation with cutting-edge generative AI without sacrificing data security. Ultimately, this use case highlights the importance of combining secure AI deployment with fairness-promoting strategies to advance equitable mental health research.

## Discussion

The integration of LLMs into healthcare presents significant opportunities for enhancing patient care and operational efficiency. However, these opportunities are coupled with risks related to data privacy and compliance. Thus, medical centers will inevitably be confronted with the need to modernize their infrastructure for leveraging LLMs safely within a controlled environment. Our Secure GPT platform addresses these challenges by providing a scalable, HIPAA-compliant solution that allows researchers to harness the power of LLMs while maintaining control over sensitive data. This centralized approach not only prevents the proliferation of insecure microprocesses but also enables equitable and fair AI development across a variety of healthcare use cases.

Our work with Secure GPT offers a blueprint for medical centers to build secure and scalable AI infrastructures that safeguard patient data while fostering clinical innovation. Without similar capabilities across institutions that enable multi-site comparisons, it remains difficult to determine the applicability and effectiveness of generative AI insights. As generative AI continues to play a more prominent role in healthcare, it is critical that institutions adopt centralized and compliant solutions like Secure GPT to ensure that sensitive information is protected and research can progress without compromise.

## Data Availability

The data underlying this article will be shared on reasonable request to the corresponding author.
